# Rings and Slings: Not Such Simple Things

**DOI:** 10.1007/s11886-022-01764-8

**Published:** 2022-10-03

**Authors:** Elyan Ruiz-Solano, Michael Mitchell

**Affiliations:** 1 Department of Surgery, Children’s Hospital Colorado, University of Colorado, Aurora, CO USA; 2 Herma Heart Institute, Children’s Wisconsin and Medical College of Wisconsin, Milwaukee, WI USA

**Keywords:** Vascular ring, Pulmonary artery sling, Kommerell diverticulum, Double aortic arch, Aberrant subclavian artery, Tracheal stenosis

## Abstract

**Purpose of Review:**

Vascular rings are congenital malformations resulting from abnormal development of the great vessels, with the consequent encircling and compression of the trachea, esophagus, or both. We conducted a review of the current literature to identify the different management strategies that can be implemented based on the prognosis of each of these anomalies.

**Recent Findings:**

Although most vascular rings occur in isolation, they can also be associated with other congenital cardiac and/or respiratory diseases; therefore, thorough investigation is necessary before definitive surgical repair. Clinical presentation varies from asymptomatic to severe, with both respiratory and digestive symptoms. Although early surgical results are acceptable, the long-term outcome is variable; therefore, there is still controversy regarding the appropriate timing of treatment. This is especially true with regard to the Kommerell diverticulum (KD) and in patients without symptoms at the time of initial surgical evaluation.

**Summary:**

As more sophisticated diagnostic tools have become available and more studies on adults affected by this condition have been published, understanding of this condition and its additional clinical implications has grown and appears to be tilting management toward earlier intervention.

## Introduction

Vascular rings account for less than 1% of all congenital cardiovascular anomalies [1]; the first observed ring was a double aortic arch, documented by Hommel in 1737 [2], Glaevecke and Doehle made the first autopsy report of a pulmonary artery sling (PAS) in 1897 [3]. However, the first post-mortem diagnosis in the clinical setting of severe bronchial compression was made by Quist-Hanssen in 1949 [4]; furthermore, the association of PAS with congenital tracheal stenosis was first characterized in 1976 by Cohen and Landing [5].

In 1787 Bayford described a post-mortem finding of a retro-esophageal right aberrant subclavian artery (ASCA) in the context of chronic dysphagia; later, Arkin named the ASCA as arteria lusoria and the term “dysphagia lusoria” was born [6–8]. In 1936, Dr. Burkhard F. Kommerell made the first radiographic diagnosis of dysphagia lusoria and esophageal compression by an aortic diverticulum in a living subject [9]. Although vascular rings do not have gender predilection, some groups report a higher prevalence of Kommerell diverticulum (KD) in women [10•].

## Embryology and Anatomy

Aortic development begins during the third week of gestation after a very intricate ontogenetic process [11]. Most aortic arch anomalies occur due to the abnormal persistence or degeneration of specific parts of the primitive aortic vascular system at a given point in embryological development [12].

The International Congenital Heart Surgery Nomenclature and Database Committee has proposed the following anatomical classification of vascular rings [13, 14]:

1. Double aortic arch
2. Right arch dominance (80%)
3. Left arch dominance (10%)
4. Balanced arches (10%)
5. Right aortic arch with left ligamentum arteriosus
6. Mirror image branching (34%)
7. Retro-esophageal left subclavian artery (65%)
8. Circumflex aorta
9. Innominate artery compression
10. Pulmonary artery sling

The two most common anatomical vascular rings are the double aortic arch and the right aortic arch with left ligamentum arteriosum [1]. The former results when both the left and right fourth aortic arches persist, forming a collar around the developing trachea and esophagus. In the latter, the left fourth aortic arch regresses, but the ligamentum arteriosum, which arises from the left sixth aortic arch, remains [15], completing the ring around the aforementioned structures.

The KD is a persistent remnant of the fourth primitive aortic arch, which has failed to regress [16]. When there is a left aortic arch with KD, the right dorsal aorta involutes proximal to the right subclavian artery (RSCA), leaving this attached to the left descending aorta through the distal portion of the right dorsal aorta. Meanwhile, in cases of right aortic arch with KD, the left aortic arch diminishes between the subclavian and carotid arteries, allowing the RSCA to arise from the remnant right dorsal arch [17]. The KD can be classified into three types based upon its pathogenesis [18]:

1. KD in the left aortic arch with right ASCA.
2. KD in the right aortic arch with left ASCA.
3. KD without ASCA.

In contrast, the distal pulmonary arterial system is normally formed from the lung buds and joins separately into the main pulmonary artery, which derives from the tronco-aortic sac [19]. The anomalous implantation of the left pulmonary artery, also known as left pulmonary artery sling, represents about 10% of all vascular rings and is associated with abnormalities of the sixth branchial arch [1]. This prevents the left pulmonary artery (LPA) from joining the main pulmonary artery, and instead the LPA courses behind the trachea to join the right pulmonary artery (RPA) [20].

## Clinical Presentation and Diagnosis

The classic clinical presentation of patients affected with a vascular ring is stridor along with a barky cough, often referred to as a “seal-bark cough.” Other frequent symptoms are recurrent respiratory tract infections, wheezing, dyspnea on exertion, and dysphagia. Some children may have apparent life-threatening events or apnea, and more rarely those with severe compression and tracheomalacia can develop critical respiratory distress requiring endotracheal intubation [21]. On the other hand, some patients with a complete vascular ring are oligosymptomatic or asymptomatic and the diagnosis is made incidentally [22, 23].

LPA sling is usually diagnosed during infancy, since these children often present with significant respiratory insufficiency. It carries a poor prognosis, and it is often associated with intrinsic congenital tracheobronchial malformations [19] such as tracheomalacia, tracheal stenosis, tracheal webs, or complete cartilaginous tracheal rings that lead to severe tracheal stenosis, which is found in more than 70% of these patients [24]. When LPA sling is also associated with additional congenital airway disease, it is often referred as a “ring-sling complex” [1]. Respiratory symptoms are caused by either external tracheal compression due to the PA sling itself or, more importantly, by intrinsic tracheal stenosis with complete cartilaginous rings. This can be life threatening, and the treatment is sometimes challenging [25].

The diagnostic work-up of patients with vascular rings has evolved dramatically in the last few decades owing to the advancement in imaging modalities, applicable not only after birth but also during pregnancy. The prenatal diagnosis of vascular rings and pulmonary artery sling begins with a fetal echocardiogram. The new American Institute of Ultrasound in Medicine guidelines for fetal echocardiography specifically direct clinicians to identify normal pulmonary arterial branching at the same time as looking for fetal arch anomalies [26]. Three-dimensional (3D) technology has gradually matured into an useful clinical tool to evaluate fetal cardiac anatomy, especially when using spatio-temporal image correlation (STIC) [27–29]. However, the quality of images for both methods is susceptible to the negative impact of certain fetal or maternal factors like fetal position and movement, oligohydramnios, or maternal obesity [30].

**Fig. 1:**
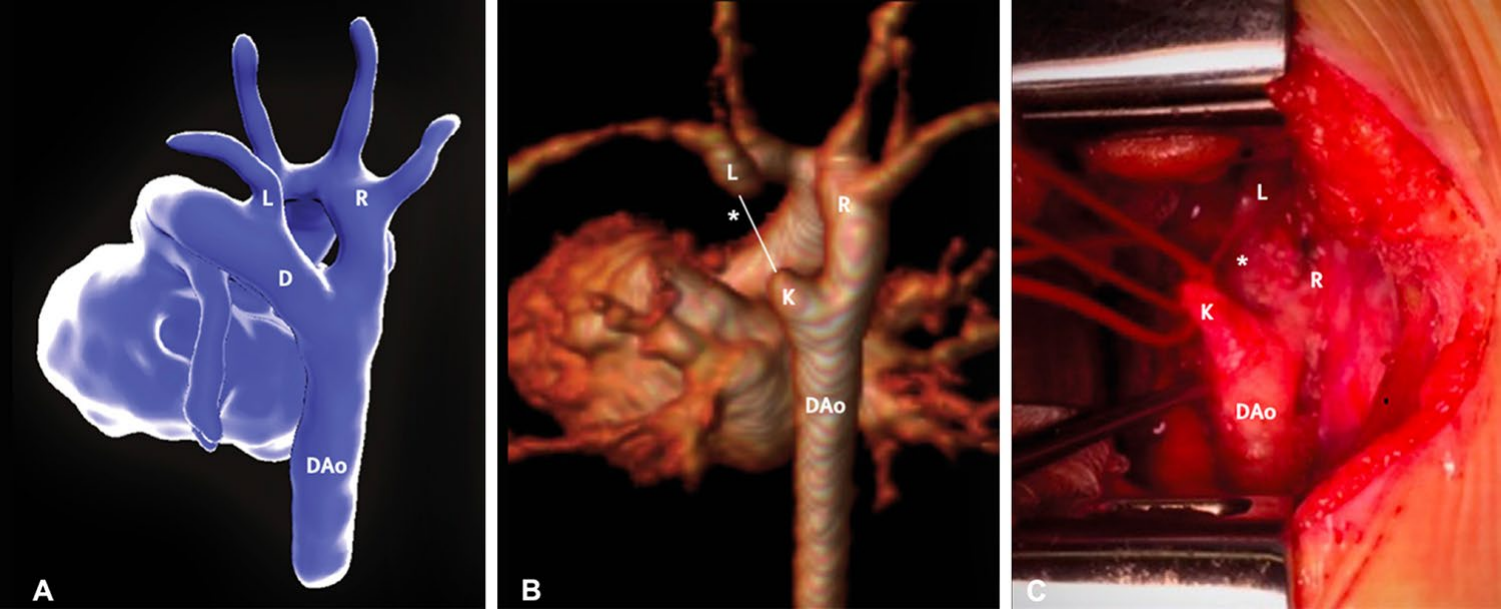
Motion-corrected MRI data from a fetus with double aortic arch at 32 weeks (**A**). Shown are the descending aorta (DAo), arterial duct (D), and left (L) and right (R) aortic arches. At 2 months postnatal age, contrast-enhanced MRI could show a right-sided arch (**B**); however, a ligamentous remnant of the left arch was predicted on the basis of the fetal MRI findings (asterisk); this finding was confirmed at surgery (**C**). The distal remnant of the arterial duct—analogous to the diverticulum of Kommerell—is also seen (K). (From: Lloyd D, et al. Lancet 2019; 393: 1619–27, with permission from Elsevier) [31]

The limitations of the fetal echocardiogram can be overcome with the use of 3D fetal cardiac magnetic resonance imaging (CMRI) with motion-corrected slice-volume registration, particularly when a reliable visualization of the fetal cardiovascular structures is required to rule out important defects of the fetal vascular system (Fig. 1) [31].

The diagnostic images for the postnatal diagnosis of patients with vascular ring range from a simple chest radiogram (CXR) to most invasive ones such as catheter angiography. The plain CXR of a symptomatic child with vascular ring will always demonstrate some abnormality [32]. The presence of a right aortic arch, right descending aorta, or focal tracheal indentation can be noted on the frontal film; while anterior bowing of the trachea, increased retrotracheal soft tissue opacification, and focal tracheal narrowing can be found in the lateral view [32, 33]. The frontal CXR mediastinal silhouette of any child with aerodigestive tract symptoms should always be carefully scrutinized despite an apparently obvious alternative etiology, such as a foreign body [34].

The contrast esophagogram or barium study was historically routinely used in the diagnosis of vascular rings and is particularly useful in patients with persistent asthma and aspiration symptoms unresponsive to standard treatment. The finding of an extrinsic pulsatile indentation is highly suggestive of a vascular ring, while its absence effectively excludes this pathology; additionally, it aids to identify other differential diagnoses such as aspiration or tracheoesophageal fistula [33, 35].

**Fig. 2:**
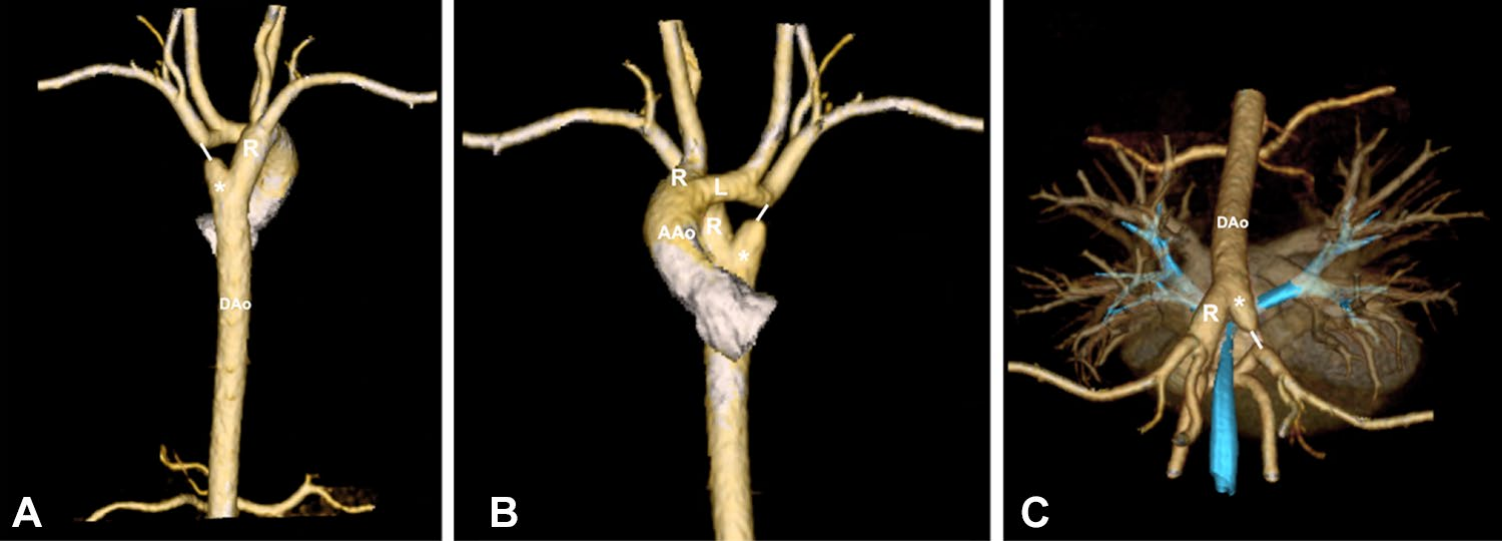
3D reconstruction of a double aortic arch. Anterior and posterior view are shown in pictures (**A** and **B**). Superior view depicts the vascular anatomy and its relationship with the trachea (**C**). Dao: Descending aorta. R, right aortic arch; AAo, ascending aorta; L, left aortic arch; asterisk, Kommerell diverticulum; white bar, ligamentum arteriosum (no captured by CT imaging)

**Fig. 3:**
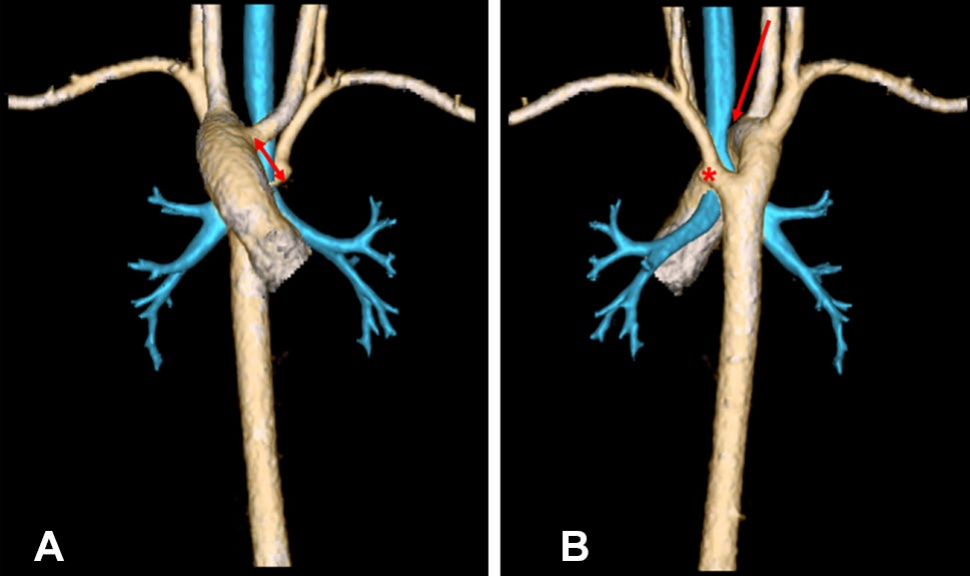
Anterior and posterior views of a right aortic arch with aberrant left subclavian (**A** and **B**). Note the significant tracheal compression (red arrow). Double red arrow: ligamentum arteriosus (not captured by CT imaging). Asterisk: Kommerell diverticulum

Although CXR and esophagogram may confirm the presence of a vascular ring, cross-sectional imaging, in the form of computed tomography angiogram (CTA) and/or MRI, is imperative to establish the exact anatomical configuration, as well as to facilitate surgical planning. It can also enable the diagnosis of other causes of fixed extrinsic esophageal or tracheal compression, such as mediastinal foregut duplication cyst [34, 36]. CTA allows multiplanar views with 3D reconstructions that offer a highly reliable description not only of the vascular anatomy but also its relationship with the trachea and the rest of the airways, as well as an accurate assessment of the lung parenchyma. Furthermore, inspiratory and expiratory CTA facilitates a dynamic evaluation of tracheal diameter, which is especially helpful in asymptomatic patients or those with associated tracheo- or bronchomalacia [35, 37, 38]; however, atretic and ligamentous structures are often not visible on contrast-enhanced imaging. CTA is a widely available tool and relatively straightforward to analyze. The time necessary to complete CTA is remarkably shorter than MRI, and therefore use of sedation is generally not necessary. Nevertheless, the need for intravenous (IV) iodinated contrast and radiation exposure may render it a less attractive option for growing and developing children (Figs. 2 and 3).

MRI studies also produce high-quality images, but do not require the use of iodinated contrast or radiation. Additionally, MRI is able to obtain cardiac hemodynamic information, making it more appealing for the pediatric population when compared to the CTA, especially for patients with additional intracardiac lesions. However, the longer study times, the need for sedation in younger patients, and the reduced spatial resolution coupled with the limited availability other than in higher level health centers are all major negative factors that make CTA the preferred imaging modality in some institutions [39]. It is important to note that regardless of the cross-sectional method implemented, both CTA and MRI have shown essentially 100% accuracy based upon surgical observation [40].

Although the flexible bronchoscopy represented a pillar in the diagnosis of tracheal stenosis of external origin in the past and was frequently used as a screening method for vascular rings prior to confirmatory cardiac catheterization, its role today is debatable [41]. Many still feel that bronchoscopy offers valuable dynamic assessment of the airway that aids not only to establish the indication and timing of repair but also in evaluating the postoperative result.

Neonatal and infant airway obstruction is of critical clinical significance. Death from airway obstruction in patients with PAS has been reported as early as 2 days of age, and it remains common before 6 months in untreated patients. Therefore, it is crucial to actively investigate for intrinsic tracheal pathology in this population, employing a combination of bronchoscopy and cross-sectional imaging. Optical coherence tomography (OCT) has also been described for allowing the detection of complete cartilaginous rings in the trachea, as well as for assessing fragmentation or dysplastic features [42].

## Surgical Management

Vascular rings are purely anatomical pathologies, and therefore no medical treatment can offer a structural correction for affected patients. Nearly all the subjects suffering from a true vascular ring will present clinical cues associated with it, and repair should not be delayed in symptomatic patients once the diagnosis is confirmed, particularly for those found to have double aortic arch and/or pulmonary artery sling. Surgery will prevent the potential complications secondary to unrepaired vascular rings later in life, such as catastrophic bleeding from indwelling nasogastric tubes, aortic dissection particularly in relation to the KD, aortic aneurysms, and persistent tracheomalacia [21]. While it is clear when to intervene on patients presenting with symptoms in the context of a vascular ring, timing is more controversial in asymptomatic cases. In an attempt to assist clinical decision-making, Worhunsky and colleagues proposed an algorithm for the management of and, prenatally diagnosed vascular rings, including asymptomatic infants [42]. Although it may not become the standard of care for many institutions, it can be used as a guide, particularly when complemented with diagnostic testing, such as dynamic assessment of the airways, to help decide when surgery is necessary. Additionally, Worhunsky’s algorithm shifts the current clinical mindset, incorporating preventive surgery among the pool of potential treatment pathways.

Gross was the first to manage dysphagia lusoria in a child by ligation of the ASCA [43]. The traditional surgical approach for repair of most vascular rings is the posterolateral thoracotomy (PLT). Pulmonary artery sling is normally approached through median sternotomy requiring, on occasion, the use of cardiopulmonary bypass (CPB). This also facilitates the direct assessment and further repair of any concomitant tracheal stenosis if necessary.

**Fig. 4:**
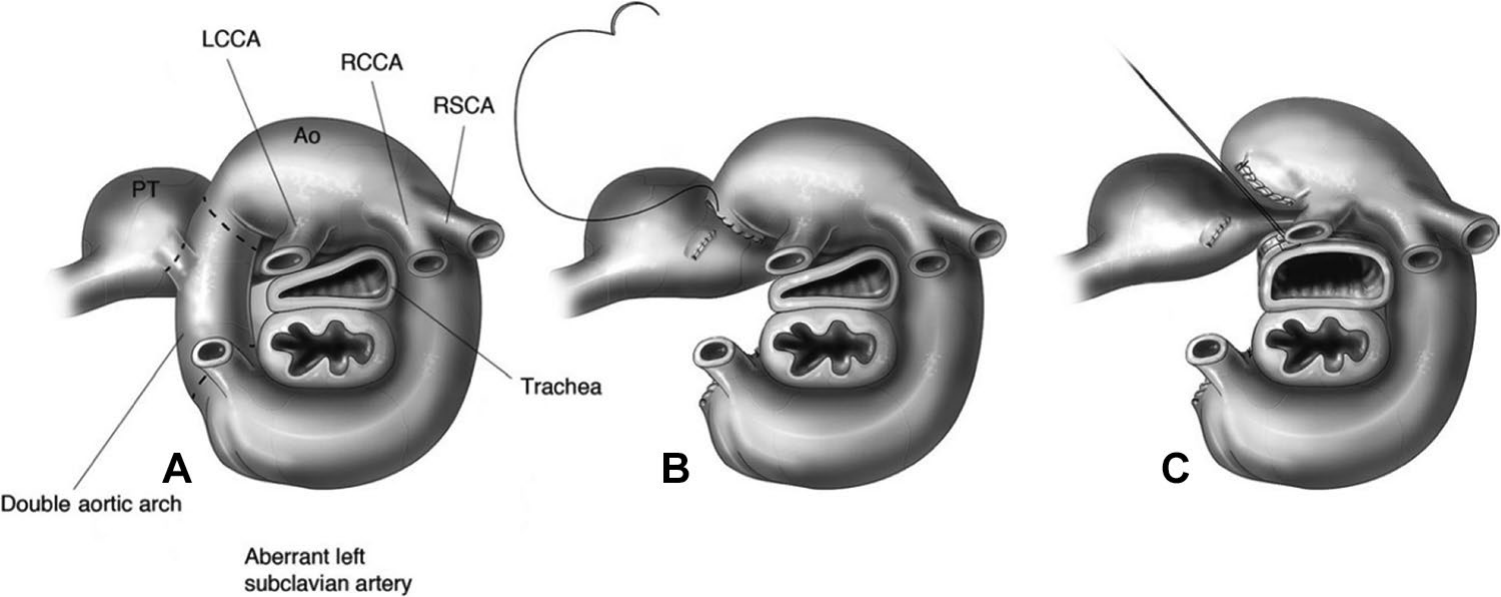
Schematic diagram of a surgical division of double vascular ring with tracheal compression associated with tracheopexy. **A**. Interrupted lines delimitating the structures to be divided (ligamentum arteriosum on the right and remanent of the left aortic arch on the right). **B**. Surgical resection of the ligamentum arteriosum and the remanent left aortic arch. Note how the trachea is compressed by the innominate artery. **C**. Anterior tracheopexy relieving the residual vascular tracheal compression. PT, pulmonary trunk; Ao, aorta; LCCA, left common carotid artery; RCCA, right common carotid artery; RSCA, right subclavian artery. (From Mitchell M. Operative Techniques in Thoracic and Cardiovascular Surgery 2011; (16:4):331–339, with permission from Elsevier) [56]

The thoracotomy side is determined by the structures needed to be resected; hence, double aortic arches with right dominance and right aortic arch with left ligamentum arteriosus or mirror image can be addressed through a left thoracotomy, while a double aortic arch with left dominance will need to be operated on from the right. In the instance of a balanced double aortic arch, it is imperative that careful analysis of the cross-sectional images occurs in order to establish which arch should be divided (Fig. 4).

The chest cavity is accessed through the third or fourth intercostal space; and after gentle lung retraction, the vascular structures of the posterior mediastinum will be visible and amenable to dissection. Posterolateral muscle-sparing (MST) thoracotomy has been associated with less pain, better shoulder function, and earlier hospital discharge in the adult population [44]. Although pain scale is more difficult to assess in children [45], MST has shown faster shoulder recovery in some series [46] without compromising surgical exposure, even in children < 1 kg of weight [44], with the promise of reducing risk of orthopedic deformation. Despite limitations, pain can be indirectly measured in children based on hemodynamic parameters, presence of irritability, and the need of comfort [45], either with administration of pain medication or physical contact.

Another surgical strategy is extrapleural access to the structures forming the vascular ring. Preserving the pleural barrier maintains intercostal muscle integrity and diminishes the risk of adhesions and development of chest wall-lung collaterals, which can be advantageous, particularly in patients with additional intracardiac lesions and/or single ventricular physiology [47]. Some groups have also reported their experience using video-assisted thoracoscopic surgery (VATS) or robotic-assisted thoracotomy [48]. In some series these procedures have shown similar results when compared to open thoracotomy, while others have described shorter in-hospital stays and reduced incidence of chylothorax with VATS [49, 50].

**Fig. 5:**
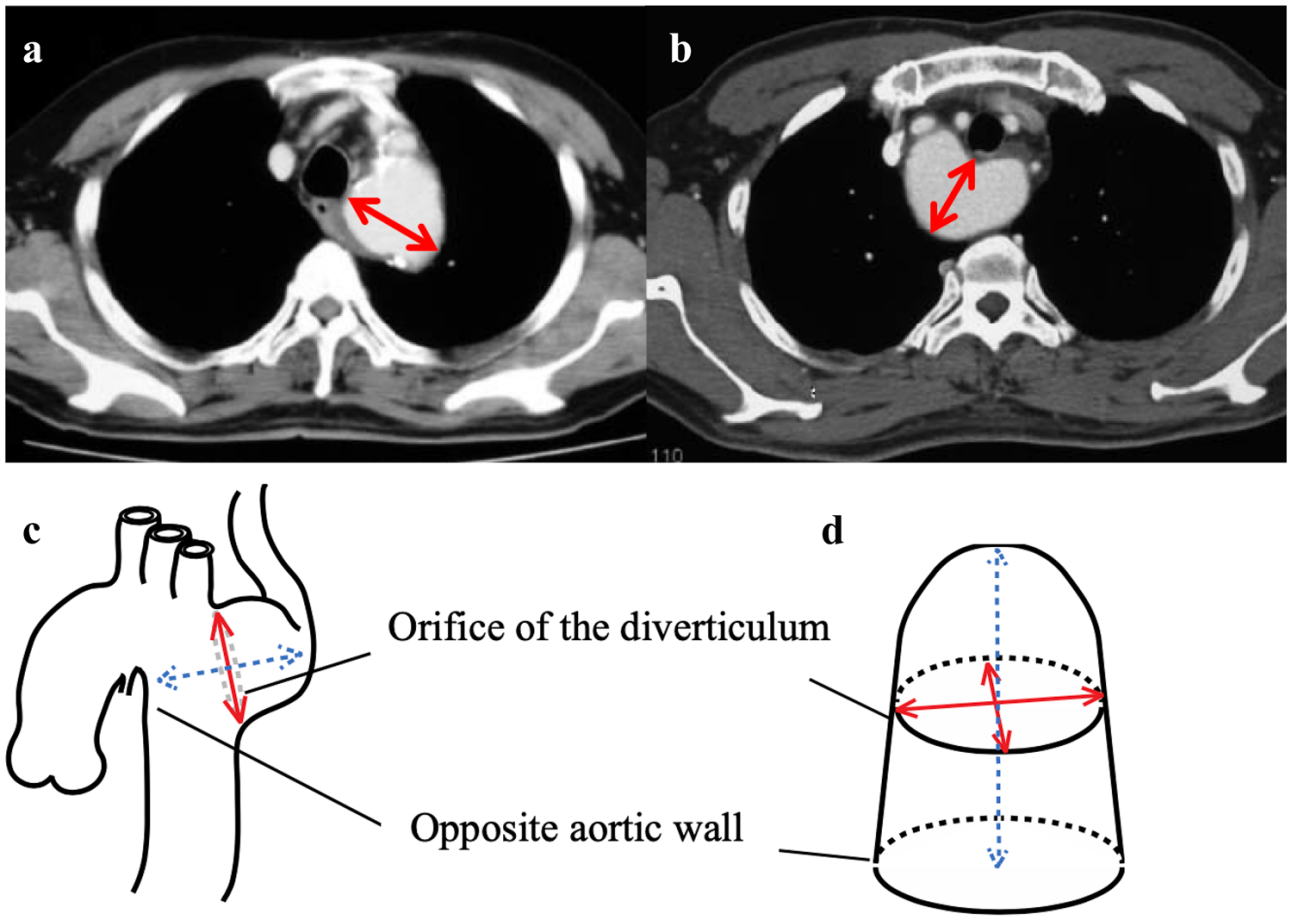
Representation of KD growth measurements including diameter in red and distance to the opposite aortic wall (DAW) in blue. Maximum distance from the diverticulum wall adjacent to the trachea to the opposite aortic wall (descending aorta) in a left aortic arch (**a**) and a right aortic arch (**b**). The two measurements proposed by Idrees et al. at the cross-sectional diameter from opposite to aortic wall to the tip of the Kommerell diverticulum (dotted arrow) and at the level of origin of the aberrant subclavian artery from the arch (solid arrow) (**c** and **d**). (From: Takanaa A, et al. Gen Thorac Cardiovas Surg (2015) 63:245–59, with permission from Springer Nature) [64]

Once the pertinent anatomic structures have been identified and carefully dissected, the ligamentum arteriosum is divided. In the case of a double aortic arch, it is important to recognize any patent segment, the origin of the supra-aortic branches, and the presence of a KD prior to transection. A KD is present in 65% of all right-sided aortic arches with left ASCA [10•] and its resection in children is recommended when its size exceeds 1.5 times the diameter of the distal subclavian artery [21]. When the subclavian artery arises from the KD, many institutions have adopted performing additional ASCA translocation to the left carotid artery [51–53]. Ota et al. were the first to introduce size criteria for KD intervention in the adult population, based upon its diameter and the distance to the opposite aortic wall (DAW). They propose surgical repair of KD to be considered for those with ≥ 30 mm diameter or ≥ 50 mm DAW (Fig. 5) [54]. For patients who have not reached these sizes, studies suggest slow growth rates of 1.45 +/− 0.39 mm/year and 2.29 +/− 0.47 mm/year for KD diameter and DAW, respectively, and therefore recommend serial CT scan follow-up for patients without symptoms [10•]. Due to the associated risk of aortic rupture and dissection at a later age, other groups advocate for a more proactive approach, with early surgical resection even in asymptomatic patients [55].

After all the vessels have been separated, it is crucial to perform a fasciolysis around the esophagus to guarantee full mobilization and release of any adhesions, hence preventing further obstruction; additionally, a tracheopexy may be necessary to ensure airway recovery and decompression [56]. Moreover, during the dissection process, it is mandatory to identify the phrenic, vagus, and inferior or recurrent laryngeal nerves, averting unnecessary manipulation to avoid neurological injuries.

The first successful repair of a pulmonary artery sling was described by Dr. Willis Pott in 1953 on a 5-month-old child performed through a right thoracotomy [57]. Although the patient survived, the outcome was complicated by complete LPA occlusion at a later time. A left thoracotomy was then introduced as a better approach once the anatomy of this lesion was better understood. Nevertheless, the use of CPB has remarkably changed the course in the care of these patients, since it allows the repair of the almost always present, significant tracheal stenosis [42].

Given the nature of the anatomical derangement, the surgical repair of the PAS comprised different sets of goals than that established for vascular rings due to malformations of the aortic arch and/or its branches. Congenital tracheal stenosis with complete tracheal rings should be addressed at the time of the repair when the stenosis is clinically severe; in that vein, some groups suggest a tracheal diameter of < 3 mm and length ratio > 60%, while others indicate a diameter: length ratio < 5.9 for tracheal stenosis as an objective measurement for indication of simultaneous surgical repair [58].

PAS repair is currently performed via median sternotomy. After the aberrant left pulmonary artery is confirmed, extensive mediastinal dissection and mobilization of the superior vena cava, main pulmonary artery (MPA), right pulmonary artery (RPA), and ascending aorta is carried out. Once the anatomic structures have been mobilized, the patient is placed on CPB, and the pulmonary vessels are isolated. The aberrant LPA is dissected to the left pulmonary hilum and subsequently divided from its origin off the RPA. This is, then, passed through from the right to the left in-between the trachea and esophagus, to finally be reimplanted anterior to the trachea into the MPA with an end-to-side anastomosis [59]. Extreme precaution should be taken not to injure the phrenic nerve; also, rotational distortion must be avoided, and the key to this is a constant check on the position of the left upper lobe division, which should be kept at its superior position until implantation. When tracheal reconstruction is needed, sliding tracheoplasty is the technique of choice due to its encouraging anatomical and clinical results [42].

## Prognosis

The natural history of unrepaired vascular rings was described by Lodeweges et al. They found that the preoperative values for peak expiratory flow (PEF), forced expiratory flow after 25% of forced vital capacity (FVC) expired, and forced expiratory flow after 50% of FVC expired were significantly lower and below normal in patients with onset of symptoms during childhood. Additionally, 8% of the patients did not show improvement after repair [60••].

Surgeries for vascular rings are generally safe operations, with a very low early and late mortality; however, additional complex cardiac and tracheal anomalies are associated with an increased risk [61]. Although most patients having a surgical repair of a vascular ring will have symptom resolution or improvement, approximately 5% will develop recurrent symptoms that are clinically significant and will require reintervention. The main causes of reoperation are obstruction of the esophagus due to KD, circumflex aorta, residual scarring, and residual tracheobronchomalacia requiring aortopexy [21].

During the last decade there has been an increasing awareness of potential underlying aortic disease associated with the KD, with medial degeneration or necrosis being the histological finding in surgically resected specimens from patients with KD in the context of aortic aneurysm or aortic dissection. It is hypothesized that the KD or remnant of the right dorsal arch is abnormal tissue rather than an innocuous aortic diverticulum, as it was programmed to involute and may have only partially done so, resulting in attenuated vascular tissue that can degenerate into an aneurysm, hence the reason to be aggressive in its resection [55].

**Fig. 6:**
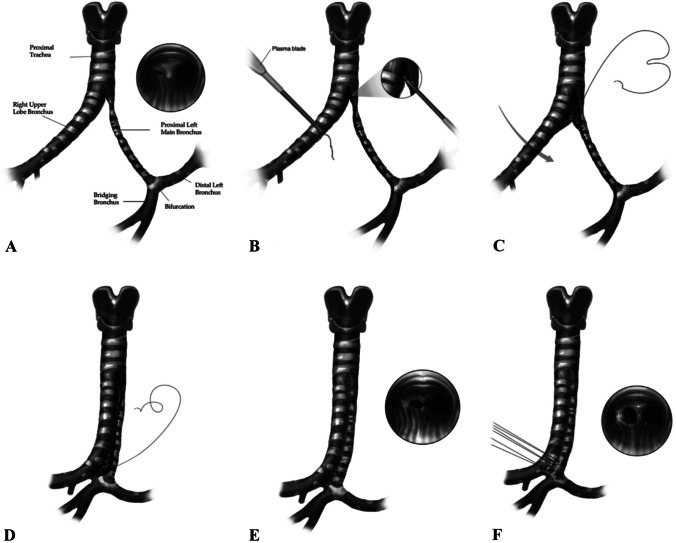
Technique of side-to-side tracheobronchoplasty. **A** Preoperative anatomy and bronchoscopy. **B** Incision is begun from the right wall of the bridging bronchus, carried out through the proximal left main bronchus, and down the left wall of the right upper right upper lobe bronchus. A strip of tissue is removed to avoid narrowing of the anastomosis in this critical region. **C** Anastomosis is performed using a running absorbable monofilament suture. **D** Anastomosis is continued onto the anterior aspect of the right upper lobe bronchus, proximal left main bronchus, and bridging bronchus, forming the neotrachea. **E** Completed trachea reconstruction with bronchoscopy demonstrating trifurcated carina and some distal malacia. If additional tracheomalacia is present, anterior tracheal suspension can be formed (**F**) under direct bronchoscopic vision to ensure optimal enlargement without distortion. (From: Ragalie WS, et al. Ann Thorac Surg 2017; 104: 666–73, with permission from Elsevier) [63]

Children with PA sling who do not require tracheal surgery have excellent clinical outcomes. Mortality in these patients is driven by the need for additional tracheal surgery, which has a combined early and late mortality of ~14% in some series [42, 62]. The introduction of the slide tracheoplasty has reduced mortality rate dramatically in some centers [62]; however, if additional intrinsic airway pathology involves distal segments, more intricate techniques are necessary to achieve a functional anatomy increasing the surgical risk (Fig. 6) [63]. The in-hospital and intensive care unit (ICU) stay are considerably longer when tracheal repair is involved as part of the operation and CPB time has been identified as an independent predictor of mortality in multivariate analysis [42]. The latter likely reflects the level of complexity of the repair, especially when PAS is associated with airways and intracardiac lesions.

## Conclusion

Vascular rings are rare congenital cardiovascular malformations whose variety of associated symptoms should prompt early surgical repair. Excluding PAS cases, operative mortality is negligible, and the vast majority of patients experience resolution of their symptoms. However, a minor proportion of repaired subjects will present with recurrence of clinically significant symptoms and require reintervention.

The presence of KD in adults with previous diagnosis of vascular ring increases the risk of aortic aneurysm and aortic dissection. As such, it is generally advisable to resect it at the time of initial operation during infancy. If unrepaired, serial follow-up imaging appears warranted.

Although being only a partial ring, the LPA sling represents a more complex spectrum of vascular rings. The complication and mortality rate of LPA sling repair are closely related to the degree of tracheal stenosis and the need for concomitant tracheal intervention. However, novel surgical techniques and implementation of a multidisciplinary approach have dramatically improved the outcomes of this cohort.
